# Sodium fluoride PET/CT with arthrography for cartilage evaluation of the knee

**DOI:** 10.1016/j.radcr.2024.02.001

**Published:** 2024-02-22

**Authors:** Alina van de Burgt, Rachèl E.L. Hezemans, Frits Smit, Menno R. Bénard, Joris A. Jansen

**Affiliations:** a Department of Nuclear Medicine, Alrijne Hospital, Leiderdorp, the Netherlands; b Department of Radiology, section Nuclear Medicine, Leiden University Medical Center, Leiden, the Netherlands; c Department of Orthopaedics, Alrijne Hospital, Leiden, The Netherlands

**Keywords:** Cartilage degeneration, Na[^18^F]F-PET/CT, Arthrography, Knee, [^18^F]-sodium fluoride

## Abstract

The presence of healthy cartilage in the knee joint, featuring smooth articular surfaces, is crucial for normal physiological knee function. However, noninvasive *in-vivo* assessment of cartilage quality in the knee remains challenging and has not been thoroughly investigated. We aimed to illustrate two clinical cases, a 62-year-old male and a 67-year-old male, presented to the orthopaedic outpatient clinic with severe knee complaints. The novel combination of sodium fluoride-18 positron emission tomography/computed tomography and intra-articular injection of a contrast agent (Na[^18^F]F-PET/CT arthrography) was performed to evaluate cartilage defects of the knee as part of a prospective patient study. The lesion size observed on the Na[^18^F]F-PET was substantially larger compared to the findings on CT. This might indicate that Na[^18^F]F-PET/CT arthrography was able to image osseous and chondral pathological changes in an early stage and in a single procedure. Na[^18^F]F-PET/CT arthrography is a promising imaging technique and might extend the diagnostic potential of nuclear and radiological imaging in the evaluation of cartilage defects.

## Introduction

The presence of healthy cartilage in the knee joint, featuring smooth articular surfaces, is crucial for normal physiological knee function. Cartilage defects in the knee affect approximately 10% of the population [1] and are detected in around 60% of all knee arthroscopies [2]. Small cartilage lesions typically do not require surgical intervention, while larger defects or unstable cartilage flaps may necessitate various surgical procedures, ranging from lavage, mechanical debridement, arthroscopic radiofrequent ablation treatment and bone marrow stimulation to tissue transplantation [3], [4], [5], [6], [7]. However, noninvasive *in-vivo* assessment of cartilage quality in the knee remains challenging and has not been thoroughly investigated.

The combination of nuclear imaging and intra-articular injection of a contrast agent (arthrography) is a promising imaging combination for the evaluation of cartilage abnormalities yielding functional information about increased bone turnover in combination with morphological details [8]. Although technetium-99m radio-labelled phosphonates can be combined with single photon emission computed tomography (SPECT/CT) to determine the uptake reference in relation to anatomical structures, positron emission tomography/computed tomography (PET/CT) offers superior spatial resolution while exposing the patient to less radiation [9,10]. In addition, sodium fluoride-18 PET/CT (Na[^18^F]F-PET/CT) facilitates the identification of increased bone turnover and provides 3D quantitative data on osteoblastic activity. While primarily used for oncological indications, Na[^18^F]F-PET/CT has been used for benign bone and joint disorders, including the assessment of subchondral bone formation of the knee [11], [12], [13], [14], [15].

Therefore, the combination of Na[^18^F]F-PET/CT with intra-articular injection of a contrast agent (Na[^18^F]F-PET/CT arthrography) might be a promising tool for noninvasive *in-vivo* assessment of knee cartilage and may provide functional information for cartilage evaluation in patients with cartilage defects and hence might guide treatment decision-making.

## Case report

### Case 1

A 62-year-old male presented to the orthopedic outpatient clinic with severe knee complaints, particularly at the posterior aspect of the left knee. A knee arthroscopy was performed after a car accident, and a medial meniscal tear was debrided arthroscopically 30 years ago. Applying force to the left knee in flexion was painful and rotation was limited. Moreover, long-distance walking was impeded and the recent exacerbation of pain prompted the patient's admission to seek further evaluation. Despite the exacerbation, the knee did not exhibit signs of warmth, swelling, or redness. Additionally, the patient had a history of hypercholesterolemia, type 2 diabetes mellitus, hypertension, and left ventricular hypertrophy. The ultrasound examination showed no Baker's cyst or other pathology observed echo graphically at the site of swelling dorsally in the left knee. Work-up revealed a corticosteroid injection for the pain. However, persistent pain necessitated further investigation and Na[^18^F]F-PET/CT arthrography was performed since the patient was considered for arthroscopic radiofrequent ablation treatment according to study protocol [16].

Na[^18^F]F-PET/CT arthrography was performed on a 5-ring Discovery MI PET/CT scanner (GE Healthcare, Chicago, IL, USA). Approximately 25 minutes after Na[^18^F]F administration (1 MBq/kg of body weight), intra-articular contrast was injected into the affected knee with an anterior approach (infrapatellar) using a concentration of 5 mL NaCl and 15 mL Ultravist 300 mg/mL. Subsequently, the patient was instructed to perform knee flexions. Approximately 35 minutes after intravenous Na[^18^F]F administration, the patient lay supine with the arms on the abdomen and the feet towards the gantry. PET data were acquired using 2 bed positions with 50% overlap and 3 minutes per bed position, with the knees centrally positioned in the field of view (FoV). Time-of-flight PET data were reconstructed using a 256^2^ matrix, 40 cm FOV, Q.Clear (beta value 700), point spread function, and CT-based attenuation correction. The diagnostic CT (120 kV, 80 mA, 1.0 second rotation time, 0.984 pitch) was reconstructed with a 30 cm FoV and a 512^2^ matrix and slice thickness of 0.625 mm. The entire Na[^18^F]F-PET/CT arthrography procedure took approximately 45 minutes to complete for the patient.

**Fig. 1:**
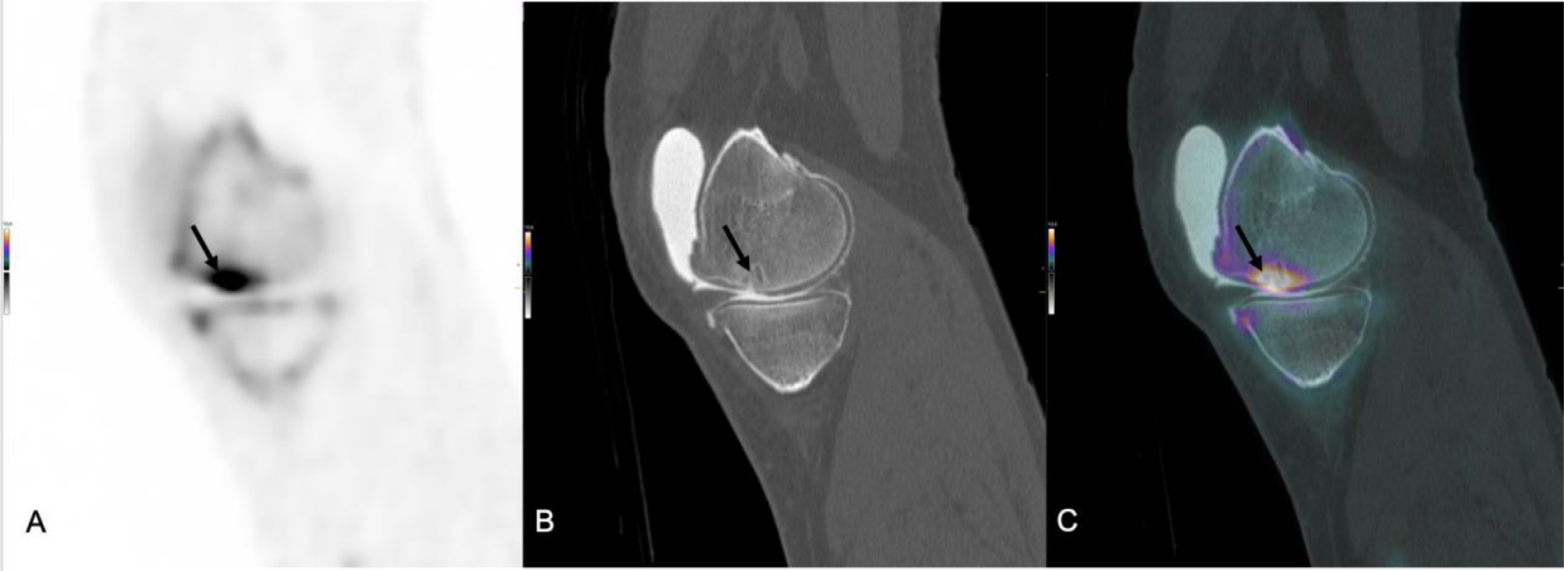
Sagittal images of a sodium fluoride-18 PET/CT (Na[^18^F]F-PET/CT) scan with intra-articular contrast (A, PET; B, CT; C PET/CT fusion) with an intense Na^18^F focus (16 × 15 mm) with Grade 4 cartilage defect, mild sclerosis, and a small subchondral cyst (arrow). Small spike-like indentations filled with contrast, consistent with degenerative cartilage, are present elsewhere in the retropatellar cartilage.

Na[^18^F]F-PET/CT arthrography showed uptake in the ventral aspect of the medial femoral condyle, corresponding to evident Grade 4 cartilage defects on CT, featuring localized sclerosis (15 x 15 mm). Dorsally, two Grade 3 and Grade 3/4 cartilage defects with increased Na^18^F uptake were noted, lacking distinct subchondral abnormalities. A medial tibial plateau cyst was observed without increased activity uptake. The minimal medial tibial plateau cartilage thickness measured less than 1 mm, although overall cartilage appeared thicker. An actively small Grade 4 cartilage defect was identified on the lateral femoral condyle, free of subchondral abnormalities. Contrast-enhanced spikes in the medial femoral and occasionally extreme lateral tibial cartilage were evident indicating degenerative changes. Retropatellar an intense 16 × 15 mm focus with Grade 4 cartilage defect, mild sclerosis, and a small subchondral cyst was visible (Fig. 1). Small spike-like indentations filled with contrast, consistent with degenerative cartilage, were present elsewhere in the retropatellar cartilage.

### Case 2

A 67-year-old male presented to the orthopedic outpatient clinic with severe knee complaints. The patient reported pain in the left knee for the past 4-5 months, which began spontaneously during walking and was associated with a feeling of impaired knee extension. The pain was localized to the medial side of the left knee and was particularly exacerbated during weight-bearing activities such as walking and sports. While night pain was initially present, it had subsided and was absent at the time of presentation in the outpatient clinic. The X-ray of the patella revealed a normal patellofemoral groove without signs of dysplasia or degeneration. This examination led to a corticosteroid injection for pain relief. However, due to persistent pain, further investigation was deemed necessary, and the patient was considered for arthroscopic radiofrequent ablation treatment [16].

**Fig. 2:**
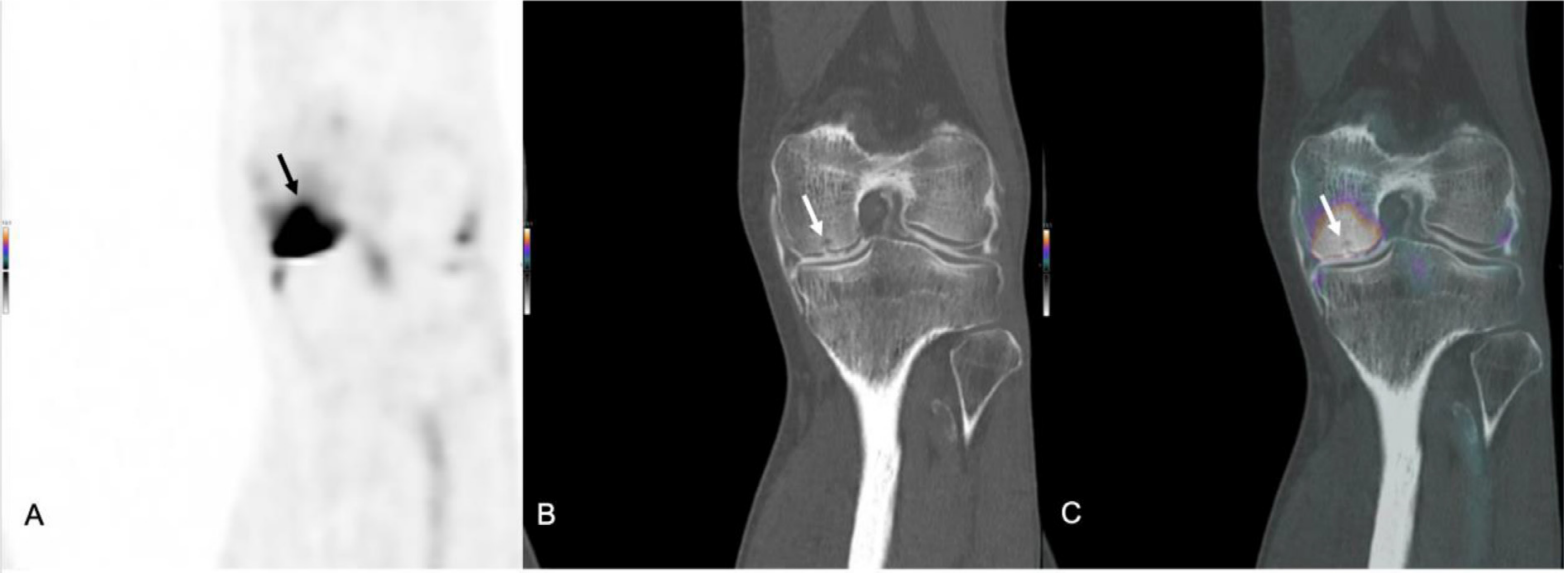
Coronal images of a sodium fluoride-18 PET/CT (Na[^18^F]F-PET/CT) scan with intra-articular contrast (A, PET; B, CT; C PET/CT fusion) with extensive subchondral cyst formation in the medial femoral condyle, accompanied by an associated cartilage defect (Grade 4) and heightened Na^18^F uptake (arrows). Osteophyte formation on the lateral side of the lateral femoral condyle, along with eminence spurring, was noted, all indicative of degeneration.

The Na[^18^F]F-PET/CT arthrography was performed following the procedure outlined in the second paragraph of case 1. The diagnostic assessment, including Na[^18^F]F-PET/CT with intra-articular contrast, revealed extensive subchondral cyst formation in the medial femoral condyle, accompanied by an associated cartilage defect (Grade 4) and increased Na^18^F uptake (Fig. 2). Osteophyte formation on the lateral side of the lateral femoral condyle, along with eminence spurring, was noted and were indicative of degeneration. The lateral meniscus was found to be intact with no evidence of a tear. There was suspicion of an oblique tear at the posterior horn of the medial meniscus. Both anterior and posterior cruciate ligaments were intact. Quadriceps tendon enthesopathy was observed, along with the presence of a Baker's cyst, and extensive vascular calcifications.

## Discussion

To the best of our knowledge, this was the first time Na[^18^F]F-PET/CT was performed in combination with intra-articular contrast for the evaluation of cartilage defects in the knee. This case report illustrated the potential advantages of Na[^18^F]F-PET/CT arthrography particularly in the assessment of osseous and chondral lesions. Furthermore, through the combination of Na[^18^F]F-PET with diagnostic CT and intra-articular contrast, the visualization of potential meniscus tears, as well as the assessment of the anterior and posterior cruciate ligaments, became feasible.

Currently, CT and magnetic resonance imaging (MRI) arthrography are clinically available tools for evaluating the condition of knee articular cartilage and assessing the stability of osteochondral defects. MRI excels in cartilage assessment and ligamentous structures, offering superior soft tissue contrast. However, MRI has limitations in detecting very early stages of cartilage degeneration [17]. In addition, CT arthrography has been reported as a highly accurate method for the evaluation of cartilage thickness compared to MRI [18]. Na[^18^F]F-PET/CT combines the advantages of CT arthrography and provides insights into bone metabolism, potentially indicating early changes in subchondral bone.

The lesion size observed on the Na[^18^F]F-PET was substantially larger compared to the findings on CT. This might indicate that Na[^18^F]F-PET/CT arthrography was able to visualize osseous and chondral pathological changes in an early stage and in a single procedure. Therefore, Na[^18^F]F-PET/CT arthrography might be effective in assessing clinically relevant chondral lesions and osseous structural alterations such as erosion, periosteal new bone formation, and ankylosis. However, the relevance of having a radiological cyst but no Na^18^F uptake remained unclear. Moreover, Na[^18^F]F-PET/CT arthrography might be a valuable alternative to MR arthrography or CT arthrography alone for the assessment cartilage defects as SPECT/CT arthrography [8]. This underscores the importance of tailoring imaging modalities to the nature of lesions, emphasizing the complementary roles of Na[^18^F]F-PET/CT and MRI in visualizing different aspects of knee pathology.

Na[^18^F]F-PET/CT arthrography is a promising imaging combination and might extend the diagnostic potential of nuclear and radiological imaging in the evaluation of cartilage defects. To demonstrate sufficient evidence for the use of Na[^18^F]F-PET/CT arthrography in clinical practice for *in-vivo* cartilage assessment, validation should take place in a prospective clinical trial, which studies a large cohort of patients.

## Patient consent

We affirm that written, informed consent for the publication of the cases has been obtained from the patients involved. The consent encompasses the dissemination of medical information, diagnosis, treatment details, and any associated images deemed relevant for publication purposes. The patients have been provided with a clear understanding of the intended use of this information, including potential publication in scientific journals, presentations, or educational materials. Assurance has been given regarding the protection of patient confidentiality, with efforts made to minimize the risk of identity disclosure. The patients have been given the opportunity to seek clarification and ask questions, and their consent has been obtained voluntarily without coercion. The right to withdraw consent at any point before publication, without any negative impact on current or future medical care, has been communicated to the patients.

Moreover, local approval to publish this study has been given by Alrijne Hospital and a medical ethics committee has given approval in accordance with local legislation.
